# Informing Facility Selection Through a Web-Based User Ratings System: Protocol for a Randomized Controlled Trial Among Mothers in Urban Lao People’s Democratic Republic

**DOI:** 10.2196/66085

**Published:** 2025-09-04

**Authors:** Amit Aryal, Emma Clarke-Deelder, Somphou Sayasone, Günther Fink

**Affiliations:** 1 University of Basel Basel Switzerland; 2 Swiss Tropical and Public Health Institute Allschwil Switzerland; 3 Lao Tropical and Public Health Institute Vientiane Lao People's Democratic Republic

**Keywords:** randomized controlled trial, health care systems, quality of care, health care utilization, eHealth

## Abstract

**Background:**

Despite the increasing options for public and private health care providers in the Lao People’s Democratic Republic (Lao PDR), choosing a high-quality provider or facility is difficult because timely and reliable information about providers is not readily available. Additionally, only 28% described their most recent visit to a health care provider as high quality, suggesting that while options for care are expanding, people may need support in finding providers that meet their quality needs. To inform efforts to improve access to high-quality care, evidence is needed on mechanisms that empower people to identify and use such care. The rapid adoption of mobile phones in Lao PDR, particularly in urban areas, offers opportunities to enhance access to timely, reliable information about health care facilities.

**Objective:**

This study aims to conduct an unblinded randomized controlled experiment using mobile phones to study whether routinely collected information on quality of care can improve access to high-quality care and patient satisfaction.

**Methods:**

Mothers with at least one child under 2 years of age who are already enrolled in the Vientiane Multigenerational Birth Cohort (VITERBI) will be invited to participate during in-person visits by the research staff. Participants will be randomly assigned in equal numbers to the control and intervention groups. The intervention group will receive a URL with facility ratings for pediatric health care services every 2 weeks via WhatsApp; the control group will not receive any messages. WhatsApp will also be used to administer biweekly surveys to both groups to assess the quality of care received in the past 2 weeks. The web page shared with the intervention group will display results from these surveys and from the pilot study. Research staff will conduct baseline and endline surveys with all participants during in-person visits, 3 months apart.

**Results:**

The trial is currently underway and scheduled for completion in 2025.

**Conclusions:**

This study will use a demand-side intervention to increase demand for high-quality child health care among mothers in Vientiane Capital. We will assess whether information on the quality of health care facilities—generated by study participants during the study period—influences mothers to change their preferred providers for nonurgent conditions.

**Trial Registration:**

ClinicalTrials.gov NCT06304831; https://www.clinicaltrials.gov/study/NCT06304831

**International Registered Report Identifier (IRRID):**

DERR1-10.2196/66085

## Introduction

While access to health care is expanding globally, there is appreciable variation in quality among providers, with low-quality care accounting for up to 5 million deaths each year [1]. These numbers are expected to grow as more people seek care and as the burden of disease shifts to complex conditions. Expanding access has also increased the options for public and private health care providers. Selecting a provider or facility, however, is difficult because timely and reliable information about providers is not readily available. In the Lao People’s Democratic Republic (Lao PDR), only 28% of people describe their recent visit to a health care provider as high quality [2], suggesting that while options for care are increasing, people may need support to find providers that meet their quality needs.

In order to inform efforts to improve people’s access to high-quality care, there is a need for evidence on mechanisms that empower people to identify and use such care. In low- and middle-income countries, the majority of efforts to date have focused on supply-side measures, such as enhancing the availability of medicines and equipment, introducing financing mechanisms that promote quality care, and strengthening the clinical competencies of health workers [1,3]. However, generating greater demand for health system quality is a key element of quality improvement strategies in low- and middle-income countries [4]. While there are new initiatives to study population perspectives and people’s care experiences, measures to “ignite demand” for high-quality care remain poorly understood.

Patients often face significant information asymmetry due to their lack of medical expertise, which prevents them from accurately assessing the quality of care they receive. This asymmetry, coupled with a lack of information about the quality of health care providers, results in suboptimal health care decisions and forces patients to rely on word of mouth or the reputation of providers [5]. Empirical evidence suggests that quality metrics can influence patient choices [6,7]. Additionally, recent studies have shown that online reviews can affect the selection of health care providers [8,9]. Therefore, given the large variation in the quality of care in low-resource settings [10,11], providing quality information can empower patients to make better-informed decisions about their health care.

We plan to conduct a randomized controlled experiment using mobile phones to study whether routinely collected information on quality of care can improve access to high-quality care and patient satisfaction. Specifically, we will examine whether participants switched providers—as a measure of improved access to quality care—based on the information provided, and whether such information led women to be more satisfied with the health care services received for their child or children. The rationale for the study is that access to the intervention (user ratings of health care facilities) can influence where caregivers seek care for their children. By providing information to caregivers across multiple quality domains that matter to users (ie, provider skill, provider respectfulness, cost, waiting time, staff helpfulness, and cleanliness), the intervention is intended to increase caregivers’ awareness of differences in quality and empower them to make more informed choices. We hypothesize that this increased awareness may lead to switching to better-rated facilities, particularly for nonurgent conditions—the study’s primary outcome. Additionally, making a more informed choice may result in greater satisfaction with the care received, due either to actual improvements in service quality or to closer alignment between expectations and experiences.

To explore these pathways, we include secondary outcomes that capture reported use of the ratings website, its perceived influence on facility selection, caregiver confidence in decision-making, and perceived quality of care. These measures will help us interpret the mechanisms through which the intervention may affect the primary outcome.

Study participants will include mothers living in 2 urban districts in Vientiane, the capital city, who have children under 2 years of age and are already enrolled in an ongoing Vientiane Multigenerational Birth Cohort (VITERBI), which includes the entire population of children born in the past 2 years in the study districts.

## Methods

### Participants and Randomization

This study consists of a randomized controlled experiment involving 660 mothers with children under 2 years of age from an ongoing VITERBI study, which includes the local population of young mothers in 4 districts of Vientiane Capital. Mothers with at least one child under 2 years old, already enrolled in the VITERBI study and living in 1 of 2 urban districts (Sikhottabong or Chanthabouly), will be invited to participate in the study during in-person visits by the research staff. We will use rolling enrollment while ensuring that each participant is enrolled in the study for a maximum of 3 months. Participants will be randomly assigned to 1 of 2 groups: (1) a control group that will not receive any information about providers, and (2) an intervention group that will receive biweekly updates on quality ratings based on reviews collected from mothers of young children in the study areas. The unit of randomization will be the individual participant. A simple random number draw, generated by the Open Data Kit (ODK) package installed on the tablets, will be used to assign participants to treatment or control with equal probability.

Research staff will administer baseline (see Multimedia Appendix 1) and endline (see Multimedia Appendix 2) surveys to all participants during in-person visits held 3 months apart. Responses to these surveys will be used to measure the study’s primary outcome: the proportion of mothers who changed their preferred health care facility for nonurgent child care services between the 2 surveys. This outcome assesses whether information about health care providers can influence users to seek alternative sources of care. For a completed CONSORT-EHEALTH (Consolidated Standards of Reporting Trials of Electronic and Mobile Health Applications and Online Telehealth) checklist, see Multimedia Appendix 3.

### Selection of Participants

#### Inclusion Criteria

All women aged 18 years or older, enrolled in the VITERBI study with at least one child under 2 years of age, living in 1 of 2 urban districts (Sikhottabong or Chanthabouly), able to read, having exclusive access to a mobile phone, possessing a WhatsApp (Meta Platforms, Inc) account, and able to understand and sign the informed consent form will be eligible to participate in the study (see Multimedia Appendix 4). The eligibility criteria will be assessed using data collected from the VITERBI study. Ability to read will be tested using the Lao script: “I use my mobile phone every day.” Before the study, we conducted interviews with 35 randomly selected women enrolled in the VITERBI study to gain insights into mobile phone ownership and usage. We found that 33 out of 35 (94%) owned a smartphone, and among these owners, all used the WhatsApp messaging app to send or receive messages and to make calls. Therefore, we limited the eligibility criteria in the main study to VITERBI participants with access to a smartphone and an existing WhatsApp account.

#### Exclusion Criteria

Women who are unwilling to sign informed consent, are under 18 years of age, do not have access to a smartphone, are unable to read the test script, or are unable to operate a smartphone will be excluded from the study.

### Study Preparation Procedures

#### Overview

There were 3 preparatory phases leading up to the pilot. Phase 1 involved a preliminary survey of smartphone ownership and use among 36 randomly selected participants in the VITERBI study. In phase 2, we conducted cognitive interviews with 5 women to ensure the understandability of the baseline and endline questionnaires, and we tested both the understandability and usability of the website design prototypes. In phase 3, we conducted a pilot study with 68 participants to test the study procedures. The final phase involved conducting the main study.

#### Phase 1: Mobile Phone Ownership and Website Development

We conducted a preliminary survey to assess mobile phone ownership among VITERBI participants, usage frequency, and the types of software used for communication. This survey included 36 randomly selected mothers from the VITERBI study residing in 2 urban districts (Sikhottabong and Chanthabouly). Given that the sample resided in the urban core of Vientiane Capital, 33 out of 35 (94%) owned smartphones, and among these owners, all were regular WhatsApp users.

**Figure 1:**
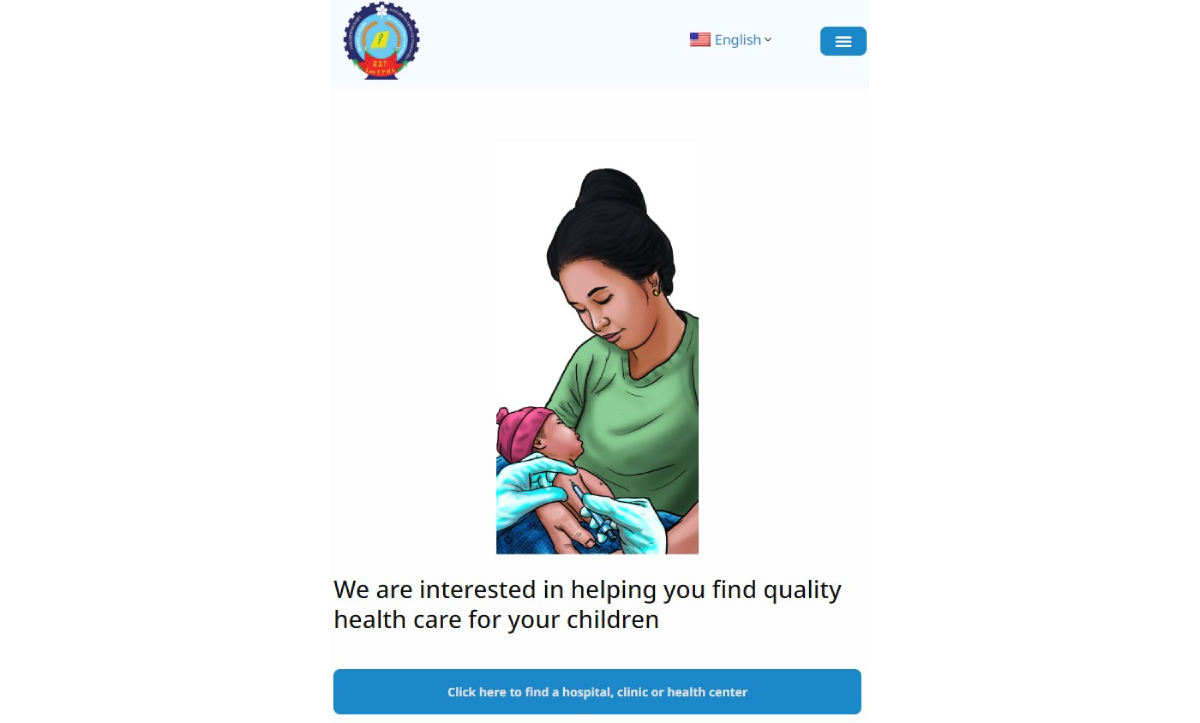
Home page of the website (intervention).

**Figure 2:**
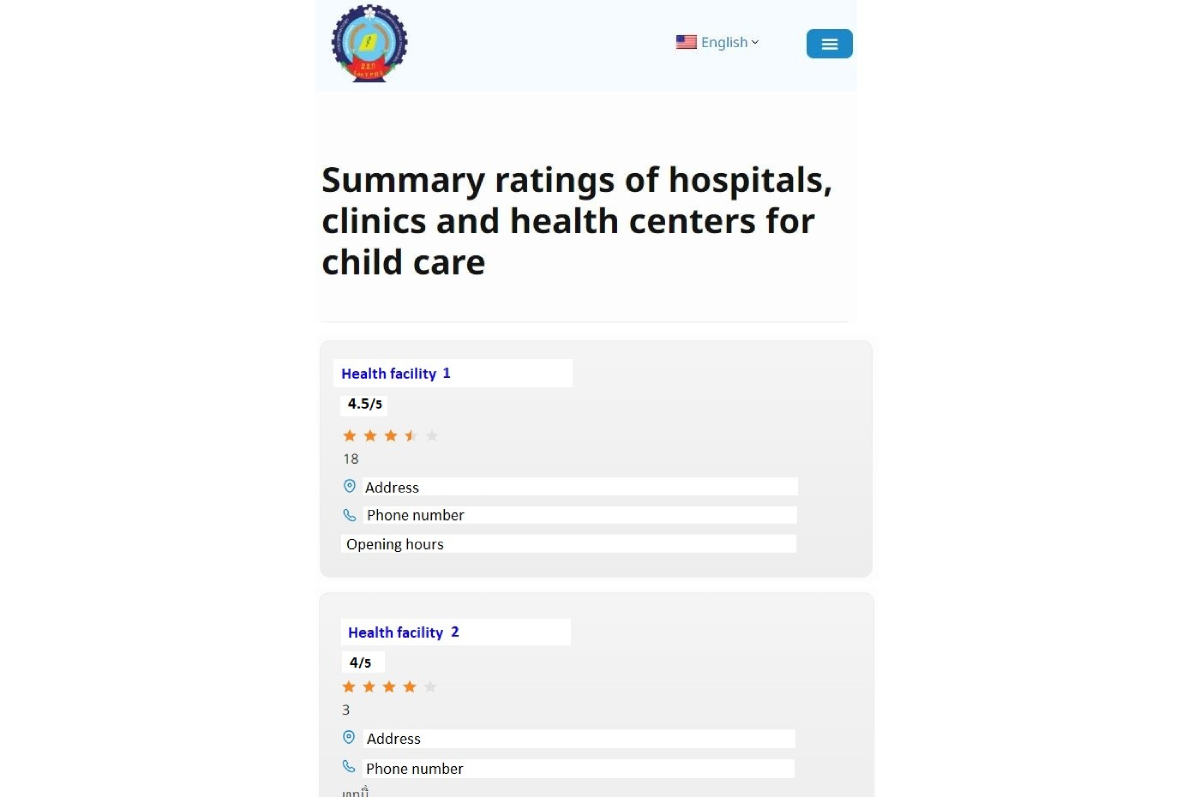
Second page of the website (intervention).

The website’s design was informed by qualitative interviews with 13 randomly selected VITERBI participants, 23 exit interviews with women at 6 public hospitals, and additional study volunteers. We used a predefined interview guide to qualitatively assess, interpret, and analyze several prototypes consisting of facility rating summaries and detailed web pages of individual health facilities. To gather additional feedback, we conducted exit interviews with mothers at the same 6 major public hospitals. These hospitals were ideal locations to test the intuitiveness and interpretation of ratings, as they also serve women from remote or rural areas who may face literacy challenges, unlike the highly educated women in the VITERBI study. The qualitative interviews revealed that mothers were familiar with ratings or scores; 1 participant described it as “similar to a score for exams in school.” Women were generally glad to receive information about health care providers for their children. There was a clear preference for using stars to represent the overall average score, and several women requested that phone numbers be displayed to help them contact health facilities. The prototypes selected for the study, shown in Figures 1 and 2, were the most favored and easily understood by the women.

The website was developed by Young Innovations, Private Limited, based in Lalitpur, Nepal, and procured by the study’s sponsor. The website and the data generated by study participants through their ratings of health facilities are jointly owned by the study’s sponsor and Lao Tropical and Public Health Institute.

#### Phase 2: Development of the Survey Questionnaire and Cognitive Interviews

Before the fieldwork in Laos, we developed baseline and endline survey questionnaires to measure primary and secondary outcomes, along with other relevant indicators. Measures of parental health literacy, parental confidence in navigating the health system, and parental confidence in interactions with health care providers were adapted from the Consumer Health Activation Index [12]. Measures related to sick children were adapted from the Integrated Management of Childhood Illnesses [13]. Additionally, measures on care utilization, care experience, expectations for health care quality, and quality of care were adapted from the People’s Voice Survey [14], which had previously been implemented in Laos in 2022 [2]. Once finalized, cognitive interviews were conducted with 5 VITERBI participants in Lao-Tai, the official language, using a combination of the “think-aloud” and “scripted probing” techniques [15]. These interviews helped identify suitable terminology for concepts such as parental health literacy, health system navigation, and confidence in interactions with care providers.

The primary outcome of the study will be based on participants’ responses to the following question, asked in both the baseline and endline surveys: “If your child is sick tomorrow with a mild fever of 38°C and cough, which hospital, clinic, or health center will you take your child to?” This question was developed in accordance with the Integrated Management of Childhood Illness guidelines [13], in which the described symptoms would be classified as nonsevere. We developed this measure specifically for this trial, guided by our theory of how the intervention might influence decision-making. We aimed to create a measure that captured intentions about where participants would seek care in a future scenario, as we anticipated that the information provided through the intervention could shape these intentions. We sought an outcome measure that could be collected from all participants without depending on recent health care utilization. For this reason, we chose this measure over alternatives such as “most recent facility visited” or “usual source of care.” Cognitive interviews and pilot surveys indicated that the measure performed well in terms of participant comprehension.

#### Phase 3: Pilot Study

We conducted a pilot study to assess the study procedures and gather feedback on the intervention and questionnaire. For the pilot’s endline survey, we added qualitative questions to capture participants’ suggestions for improving the website. Based on pilot results, we made minor revisions to the baseline and endline survey questions to enhance clarity. We also refined the quality components to overall quality, skill and experience of the health care provider, equipment, respectfulness of the health care provider, cleanliness, waiting time, and cost.

### Study Procedures

Research staff, with support from the respective village offices, will recruit participants during in-person visits. At the first meeting, before enrollment and the baseline survey, research staff will explain the study and obtain informed consent. The informed consent will cover key information, including the study’s objectives, duration, level of participant involvement, and the assurance that participation is entirely voluntary. Tablets equipped with ODK will be used to record responses from enrolled participants during the baseline and endline surveys. All participants will receive a small gift in appreciation of their participation, in keeping with customary practice in Lao PDR.

After completing the baseline survey, research staff will assist all enrolled participants in connecting with the study’s WhatsApp number and will demonstrate how to submit biweekly user ratings of facilities. In addition, staff will encourage participants in the intervention group to use the website, as needed, to access information on pediatric health care facilities and view user ratings submitted by their peers. Research staff will demonstrate the 3 main pages of the website, show participants how to navigate between them, and answer any questions they may have. The study coordinator will record enrolled participants weekly, along with their group assignment (intervention or control), and will schedule dates for each participant to receive biweekly surveys, the intervention URL (for those in the intervention group), and the endline interview.

Following enrollment, all participants will be asked to rate or review the most recently visited health care facility every 2 weeks; these ratings will generate user scores for the website (intervention). Participants will receive a WhatsApp message from the study coordinator: “Did you visit a health center, hospital, or clinic for your child in the last 2 weeks?” Those who respond “yes” will receive a URL to a web-based survey, along with their study ID and the last 4 digits of their phone number. Before completing the survey, participants will be required to enter their study ID and the last 4 digits of their phone number. This step will help track survey completions and identify duplicate entries. Once the 2 fields are entered, participants will be asked to complete the survey by identifying the name of the health facility visited, rating the quality of care received, and providing any additional comments about their visit. The questions in the web-based survey are adapted from the People’s Voice Survey questionnaire [16], a mobile-based tool designed to capture patient experiences. All questions use multiple-choice response options and focus on participants’ most recent visit to a health care facility within the past 2 weeks. Participants are asked to assess the quality of care their child received by responding to the following items:

- How would you rate the overall quality of care your child received?
- How would you rate the knowledge and skills of your provider?
- How would you rate the equipment and supplies available to the provider, such as medical equipment or access to laboratory tests?
- How would you rate the level of respect your provider showed you?
- How would you rate the amount of time you waited before being seen?
- How would you rate the courtesy and helpfulness of the health care facility staff, excluding your provider?
- How would you rate the cost of services?
- How would you rate the cleanliness of the facility?

For each question, participants will select one of the following response options: excellent, very good, good, fair, poor, “I do not know,” or “I refuse to answer.”

The principal investigator will monitor survey entries weekly to ensure completeness and identify duplicate entries. In the case of duplicates, the most recent entry will be retained. If participants do not respond to the initial WhatsApp message or fail to complete the mobile survey, the study coordinator will make up to 5 attempts to contact them via WhatsApp and administer the survey over the call.

Average ratings for overall quality of care and for specific dimensions of care will be displayed on the web page, which is the main component of the intervention. The study coordinator will share the URL of the health facility ratings website with the intervention group biweekly via WhatsApp. The message will read: “Sabaidee, please click on the link to see ratings of hospitals, clinics, or health centers for health care for your children, and let us know if you have any questions or comments.” The control group will not receive any intervention. Given that all participants in the pilot study exchanged WhatsApp messages at least once per month, biweekly sharing of the intervention URL over 3 months is expected to provide sufficient exposure to the website for participants in the intervention group. Research staff will conduct endline surveys with all participants during in-person visits 3 months after the baseline survey.

### Description of the Study Intervention

Every 2 weeks, the intervention group will receive a URL via WhatsApp linking to the website displaying user ratings of health care facilities that provide pediatric care. Although the survey is short, we were concerned about potential survey fatigue and, therefore, limited study participation to 12 weeks (6 survey rounds).

**Figure 3:**
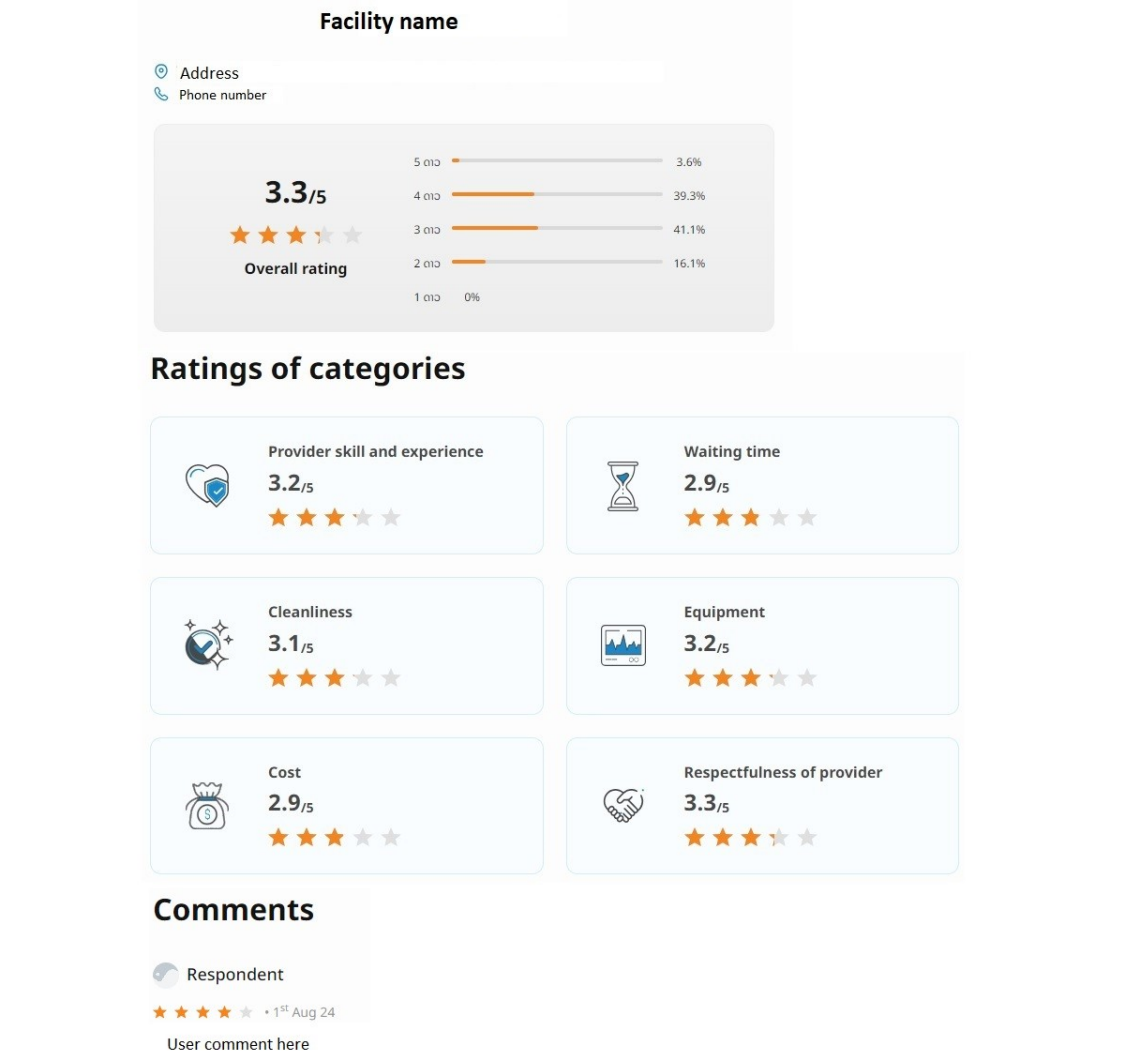
Third page of the website (intervention).

The website will consist of 3 unique pages, as shown in Figures 1-3, and will be accessible to anyone with an internet connection. All text will default to Lao, the official language of Lao PDR, while an English version will also be available for demonstration purposes. The logo of the Lao Tropical and Public Health Institute will be displayed at the top of the facility ratings website.

The website will consist of 3 unique pages: the home page, a listing of health care facilities (second page), and a detailed review of a single facility covering multiple quality components (third page). Figure 1 shows the home page. Figure 2 displays the list of health care facilities, with those having the highest average overall rating shown at the top. Only facilities with at least three reviews will be listed, while those with fewer reviews will be hidden. Clicking on any health facility will display reviews and average ratings for multiple quality components of that facility—provider skill, respectfulness of the provider, cost, cleanliness, equipment, and waiting time—along with comments left by recent users (Figure 3). The website’s design will remain fixed for the duration of the study; however, the ratings will be updated as participants submit new entries.

Health facility reviews will be generated based on biweekly reports of recent visits by all participants. Participants will receive a WhatsApp message asking whether they have visited a health care facility for their child in the past 2 weeks. Those who respond “yes” will receive a link to complete a short survey to review or rate their experience. The overall quality rating, which determines the order of facilities on the website’s second page (see Figure 2), is calculated based on participants’ responses to the question: “How would you rate the overall quality of the health facility?” Participants are asked to select 1 of the following response options: “excellent,” “very good,” “good,” “fair,” or “poor.” The average rating is calculated using numerical values assigned to these responses, with scores ranging from 5 (excellent) to 1 (poor). Golden stars visually represent the overall average quality rating. To further assist participants in exploring health facilities, hyperlinks to each facility’s address and phone number are provided, along with their opening hours.

Figure 3 shows the average ratings across multiple quality domains for a selected health facility, along with comments on participants’ experiences. Participants can access this information by selecting the facility’s name on the website’s second page (Figure 2).

### Auditing

No audits are planned. The principal investigator will be in Lao PDR during enrollment, baseline, and endline surveys. While in Lao PDR, he will monitor the progress of enrollment and survey responses daily and will remotely oversee the progress of mobile phone surveys every week. His review will include checking the progress and accuracy of enrollment logs, versions of enrollment logs, and the completeness of baseline and endline survey responses. Additionally, he will supervise the weekly web-based surveys that collect quality ratings of health facilities and ensure the functionality of the website.

### Interim Analysis and Stopping Guidelines

Interim analyses are not planned for this study. Enrollment will stop once 660 participants have been recruited. Participants may withdraw from the study at any time. If the study team is uncertain whether a participant intends to withdraw, they may attempt to contact her up to 3 times at different times of the day. If she remains unreachable, the study team will conclude her participation.

The study team may discontinue a survey with an individual participant if she is not reachable after 3 attempts or if she has not responded to consecutive mobile survey questions. The project sponsor may terminate the study prematurely under certain circumstances, including ethical concerns, insufficient participant recruitment, or an inability to continue data collection due to public health emergencies.

### Study Design

**Figure 4:**
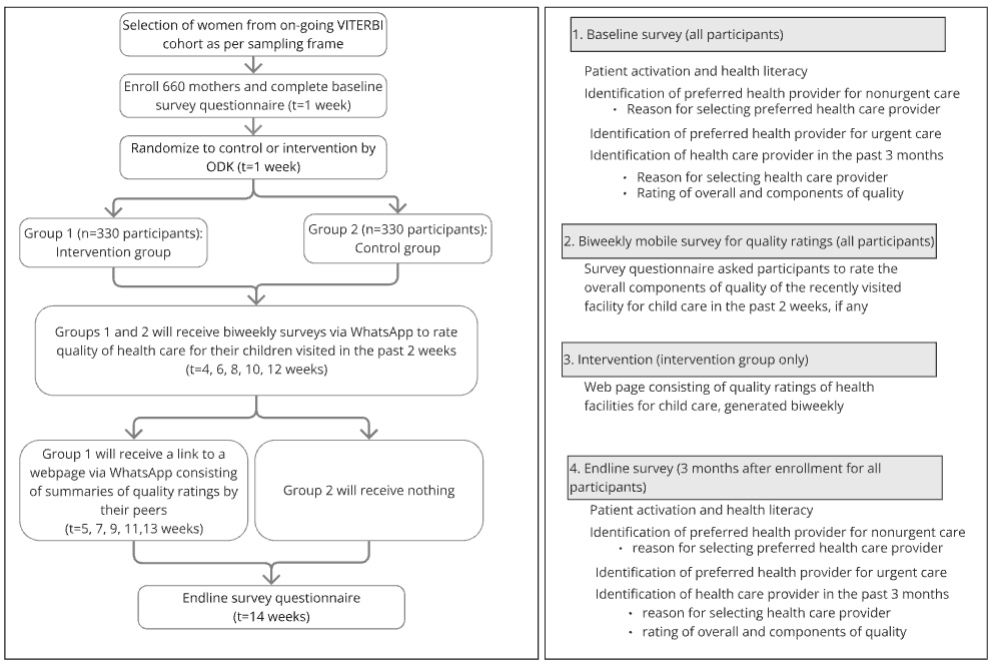
CONSORT (Consolidated Standards of Reporting Trials) study flow diagram.

This study is an unblinded randomized controlled experiment with rolling enrollment. Participants’ preferred facility for nonurgent care will be recorded during the baseline survey, immediately following enrollment. A detailed overview of the methodology and timing of the intervention is shown in Figure 4.

### Sample Size Determination

The required sample size was calculated based on the proportion of mothers who changed providers for nonurgent care in a 6-week pilot study involving 58 participants. In the pilot, all participants received the intervention—a web page of facility ratings—once or twice, depending on when the endline survey was scheduled. A conservative sample size was calculated to detect a 12-percentage-point change in the outcome with 80% power and α=.05; 330 participants per group would be required. Assuming 10% attrition, the total required sample size was set at 660 participants.

### Randomization

Participants will be randomized using the ODK package installed on tablets at enrollment. A simple random number generated by ODK will assign participants to the treatment or control group with equal probability.

### Blinding

This will be an “open-label” trial; however, the analyst will be blinded during the endline assessments.

### Adverse Events

We do not anticipate any adverse events resulting from this study. Participants will receive, via WhatsApp, a URL linking to the website containing quality ratings of health care facilities for children in Vientiane Capital.

### Unexpected Events

We have developed study procedures and a detailed calendar to ensure a shared understanding of key activities and their timelines. These measures are also designed to minimize the impact of unexpected events, such as staff changes. A journal will be maintained to track critical events, including website downtime.

### Equipment

We do not anticipate the need for additional equipment. Participants will use their own mobile phones to communicate with the research team.

### Attrition

We attempted to minimize loss to follow-up by excluding participants who permanently or temporarily moved away from the 2 study districts and by monitoring participant engagement throughout the study period. The study coordinator called participants who did not respond to the biweekly surveys to encourage participation and resolve any issues they may have been experiencing with accessing the data.

### Data Management

Survey data will be recorded using unique identifiers for each participant. An enrollment log will be maintained to link these codes with participants’ phone numbers and will be used solely for study administration. Responses to the baseline, endline, and mobile-based surveys will be stored electronically using only the individual codes, without any phone numbers.

AA and a research coordinator will maintain the enrollment logs electronically on a password-protected, secure Swiss TPH server that will be regularly backed up. The enrollment log will be saved using the following format to ensure traceability of any changes: LaosMR_enrol_v1.0_DD.MM.YYYY. The research team (AA, GF, and ECD) will have access to password-protected survey responses from the baseline, endline, and mobile phone surveys.

Data collected during the biweekly surveys will be stored on a password-protected cloud server, accessible only to the research team (AA, GF, and ECD). Hard copies containing participant information will be kept in a locked filing cabinet in the project office at the Lao Tropical and Public Health Institute. These files will be accessible only to the local study coordinator and enumerators responsible for conducting the baseline and endline surveys.

We will use WhatsApp for 2 main purposes: (1) to ask all participants whether they have visited a health care facility for their children in the past 2 weeks and, if they respond affirmatively, to send them a link to an online survey to rate the quality of care they received; and (2) to provide the intervention group with a URL link to the website displaying user ratings of health facilities for pediatric care. The local study coordinator will communicate with participants using a dedicated tablet and a unique WhatsApp account, used exclusively for the study. The tablet will be password-protected and accessible only to the local study coordinator. Messages exchanged between participants and the study coordinator on WhatsApp are secured with end-to-end encryption [17]. At the conclusion of the study, all contacts will be deleted, and the WhatsApp account will be disabled.

### Outcomes

Trial outcomes will be measured through the in-person endline survey. For the primary analysis, we will assess the proportion of mothers who changed their preferred health care facility for nonurgent child care services between the baseline and endline surveys. The null hypothesis is that there is no difference between the control and intervention groups. The alternative hypothesis is that there is a difference between the 2 groups. This hypothesis aligns with our study aims, as it examines whether the use of provider facility ratings influences caregivers to seek alternative sources of health care for their children. Our primary analysis is cross sectional; for the panel analysis using the care-seeking panel, observations will be clustered at the household level.

Nonurgent care is defined as a child presenting with a mild fever of 38°C accompanied by a cough. During the baseline and endline surveys, participants will be asked to specify their preferred location for child care using the following question: “If your child is sick tomorrow with a mild fever of 38°C Celsius and cough, which hospital, clinic, or health center would you take your child to?” The primary outcome will be a binary indicator reflecting whether participants changed their preferred location between baseline and endline.

We used the proportion of mothers who changed providers (n=26, 45%) for nonurgent care in a 6-week pilot study involving 58 participants. All participants in the pilot received the intervention—a web page of facility ratings. Eligible women will be randomly selected from the ongoing VITERBI study for enrollment, and recruitment will continue until we reach 660 participants, with 330 in each intervention arm, assuming 10% attrition. Enrolling 300 participants per intervention arm provides 90% power, with a 2-sided α of .05, to detect a 12-percentage-point change in health facility choice, assuming 80% follow-up. We will collect and analyze baseline and endline survey data from mothers enrolled in the study. We anticipate completing 600 endline surveys and analyzing this sample.

Additionally, we plan to measure 6 secondary outcomes to explore further effects of the intervention. These relate to caregivers’ actual health care use over the past 3 months, including satisfaction with care received, frequency of health care service use, and changes in the facility used for their most recent visit. Additionally, the secondary measures capture whether caregivers selected health facilities based on online or digital information, whether they changed their preferred provider for urgent care, and their confidence in identifying the best facility for their children’s care. The secondary measures are presented in Textbox 1.

**Secondary measures.**

**1. Proportion of mothers satisfied with health care for their children during their most recent visit (time frame: 3 months)**

This secondary outcome measure captures mothers’ satisfaction with the health care facility visited most recently for care of their child under age 2 years. This measure will be available only for mothers who sought care for their child during the study period. It will be a binary variable, with satisfaction defined as responding “excellent” or “very good” (as opposed to “good,” “fair,” or “poor”) to the question: “How would you rate the overall quality of care you received?” referring to the most recent visit for their child under age 2 years within the past 3 months.

**2. Number and type of health care providers used in the past 3 months (time frame: 3 months)**

This secondary outcome captures the number and types of providers (government health centers, government hospitals, private clinics, and private hospitals) visited by mothers for their children’s health care during the study period. Every 2 weeks, all enrolled participants will be asked via a mobile app: “Did you visit a health center, hospital, or clinic for your child in the last 2 weeks?” Participants who respond “yes” will be asked to complete an online survey about the visit, specifying the health facility they visited.

**3. Proportion of mothers who changed their most recently visited health care provider for children (time frame: 3 months)**

This secondary outcome measures the proportion of mothers who changed the health care facility they most recently visited for child care services between the baseline and endline surveys. This will be a binary variable and will be available only for caregivers who sought care during the 3 months preceding both the baseline and endline surveys.

**4. Proportion of mothers choosing the preferred facility for nonurgent care based on online or digital information sources (time frame: 3 months)**

This secondary outcome measures the proportion of mothers who used online or digital information sources to inform their choice of providers for nonurgent care. During the baseline and endline surveys, participants will be asked to specify their preferred location for nonurgent child care using the question: “If your child is sick tomorrow with a mild fever of 38°C and cough, which hospital, clinic, or health center would you take your child to?” Immediately following this, participants will be asked: “Was this choice informed by online or digital information services?” This outcome will be recorded as a binary variable.

**5. Proportion of mothers who changed their preferred health care provider for children for urgent care (time frame: 3 months)**

This secondary outcome measures the proportion of mothers who changed their preferred health care facility for urgent child care services between the baseline and endline surveys. Urgent care is defined as a child presenting with a very high fever and a red rash covering the body. During the baseline and endline surveys, participants will be asked to specify their preferred location for urgent child care using the following question: “If your child is sick tomorrow with a very high fever of 39-40°C and a red rash covering their body, which hospital, clinic, or health center would you take your child to?”

This outcome will be a binary variable indicating whether participants changed their preferred location for urgent care between the baseline and endline surveys.

**6. Proportion of mothers confident they can identify the best place for care for sick children (time frame: 3 months)**

This secondary outcome measures the proportion of mothers who feel confident in identifying the best place to seek care for their sick children based on the child’s health problems. During the baseline and endline surveys, participants will respond with “strongly agree,” “agree,” “unsure,” “disagree,” or “strongly disagree” to the following statement: “I am able to identify the best place to get care for my child when he/she is sick.” This outcome will be recorded as a binary variable: participants responding “strongly agree” or “agree” will be classified as confident, while those responding “unsure,” “disagree,” or “strongly disagree” will be classified as not confident.

### Statistical Analysis

We will use the STATA (StataCorp) statistical software package for all analyses. Descriptive statistics will first be used to assess differences between the intervention and control groups in terms of baseline characteristics and potential confounding variables. Missing covariates will be imputed as needed, and system use (including adherence to the intervention) will be measured through self-reports at endline. Differences between the intervention and control groups after the intervention will then be analyzed for both primary and secondary outcomes. Two-tailed *t* tests or ANOVA will be used for continuous variables, and chi-square tests will be used for binary outcome variables.

For our main analysis, differences between the 2 arms (participants who received provider information biweekly and those who received no information) will be assessed using regression models. Participants with missing outcome data will be excluded from the analysis, as we do not plan to impute outcomes. For missing baseline covariates, we will assume that the data are missing at random and conduct a complete-case analysis using only observations with complete data for the relevant variables. Additionally, we will perform multiple imputation to handle missing responses for baseline covariates.

Given that this is a randomized trial with individual-level randomization and a cross-sectional design, our primary analysis will compare outcomes between treatment arms using data measured at endline. Therefore, we do not anticipate issues related to clustering or intraindividual correlation in the analysis of primary outcome measures. Treatment effects for continuous outcomes will be estimated using linear regression with robust SEs to account for potential heteroskedasticity, using the following regression model:

*Y_i_* = *β*_0_ + *β*_1_*T_i_* + *e_i_*

where *Y_i_* is the outcome for individual *i*; *T_i_* is the indicator for the treatment group; *β*_0_ is the value of the control group; *β*_1_ is the estimated treatment effect; and *e_i_* is the error term. For binary outcomes, we will use the following regression model:

logit(*Y_i_*) = *β*_0_ + *β*_1_*T_i_* + *e_i_*

In secondary analyses, we will adjust for baseline covariates—including age, wealth status, educational level, ethnicity, and parental health literacy—to account for potential differences and address any imbalances between the treatment and control groups.

### Ethics Considerations

This study was conducted in accordance with the Declaration of Helsinki and received ethics approval from the Ethikkommission Nordwest- und Zentralschweiz in Switzerland (approval ID AO_2023-00063) on September 8, 2023, and from the National Ethics Committee for Health Research (NECHR) in Lao PDR on February 6, 2024 (No. 16/NECHR). The research will adhere to the principles of the Helsinki Declaration, and any secondary use of the study data will undergo new ethical approvals.

All study participants provided informed written consent before taking part in any study procedures. They were informed that participation was voluntary, that they could withdraw at any time, and that withdrawal of consent would not affect their subsequent medical assistance or treatment. No financial compensation was provided for participation; however, a small gift of appreciation was offered upon successful completion of the endline survey.

The project included several steps to safeguard data privacy. First, deidentified survey responses (baseline, endline, and mobile) were stored on password-protected servers. Second, WhatsApp was used only as a communication channel with participants and not for any data collection purposes. Communication was managed by dedicated staff using a unique WhatsApp profile created specifically for the study. All exchanges via WhatsApp were protected by the platform’s default end-to-end encryption. Finally, to further minimize risks, WhatsApp records were deleted once participants had completed the endline survey.

## Results

The trial is currently in progress and is expected to be completed in 2025. From the results, we aim to determine whether web-based reviews of health care providers can influence mothers’ care-seeking behavior for their children in Vientiane, an urban setting in Lao PDR. Additionally, we aim to validate a scalable model for generating demand for high-quality care in urban, low-resource settings with high mobile phone coverage. Findings will be disseminated through local seminars, international conferences, and peer-reviewed journals.

## Discussion

### Anticipated Findings

This protocol describes a randomized controlled trial designed to test whether a demand-side intervention influences health facility choices among mothers in Vientiane capital. We will assess whether information about the quality of health care facilities—generated by study participants during the pilot and study periods—encourages mothers to change their preferred providers for nonurgent conditions. Additionally, we will assess whether the intervention leads to higher satisfaction with care at the most recently used facility and test our theory of change that mothers in the treatment group are more confident in their ability to identify the best place to seek care for sick children.

This protocol has several key strengths. It will contribute to the literature on how to “ignite demand” for high-quality care, as called for by the Lancet Commission on High-Quality Health Systems. This study is among the first published efforts to generate demand for high-quality care, providing evidence on how users of the health system can influence its quality. The results will apply to health conditions beyond the focus of this study—health care for children. The study is particularly relevant in Lao PDR, where the number of health care providers is rapidly growing, ranging from small primary health centers to large tertiary hospitals. The absence of effective gatekeeping allows people to seek care at any facility, including tertiary hospitals. Despite the growing care options, Laotians remain unsatisfied with the quality of care, with only 28% rating their usual health care provider as high quality. It is critical to understand and address sources of low-quality care, as low perceptions of care quality can lead people to forgo care or not adhere to medical advice [18].

A recent study highlighted that facility selection is influenced by factors such as illness severity, distance, technical skills and respectfulness of health care providers, cost, availability of equipment and medicines, waiting times, and informal payments [19]. Many of these factors, however, are not readily accessible or comparable across facilities, despite persistent dissatisfaction with care quality and untapped options. This study seeks to change the status quo by empowering patients with comparable information on health care quality across multiple facilities.

Through online ratings of health care facilities for children, we aim to study whether access to information about providers influences caregivers’ choice of facility—measured by switching behavior—as an indicator of improved access to quality health care. We will also assess whether this information affects caregivers’ satisfaction with the health services received for their child or children. Drawing on findings from previous studies [2,19], the website presents user ratings across quality domains valued by residents of Vientiane, including provider skills, respectfulness, cost, availability of equipment, waiting time, cleanliness, and helpfulness of support staff.

As one of the first studies of its kind in a low-resource context, we aim to evaluate the online platform’s perceived usefulness, gather participant feedback, and measure engagement metrics such as page views and reported use of the website. In addition, we will assess whether the website increased caregivers’ confidence in selecting a health care provider for their children.

While empowering patients with information to make informed care choices, the intervention could also have unintended consequences. For example, demand for tertiary hospitals—already burdened with long waiting times—might increase, as they are generally perceived to provide the highest quality care. This effect may not apply to all types of illnesses, particularly those that can be easily diagnosed and treated at smaller facilities. Additionally, private care providers could attract patients by improving observable aspects of care without addressing underlying gaps in technical quality.

Despite potential unintended consequences, our study offers a demand-side approach to addressing low satisfaction with care quality by empowering patients with actionable quality metrics. We aim to validate a scalable model for “igniting” demand for high-quality care in low-resource settings by establishing an accountability mechanism that links provider performance with patient preferences.

### Limitations

This protocol also has some limitations. First, the study may inadvertently encourage bypassing of lower-level public health facilities, directing mothers toward specialty hospitals or private providers. A recent study showed that a large majority (64%) of people used public hospitals as their preferred health care provider, while only 13% used health centers [20]. As a consequence of the study, government-managed specialty hospitals and private facilities may experience increased demand for services for minor health conditions that could be better managed at lower-level government facilities. This shift toward private and specialty providers could raise out-of-pocket expenditures for health care clients and increase costs for the health system as a whole. Second, the quality of care measures used in the study are based on participants’ perceptions and may be influenced by social desirability bias. However, previous studies have shown that people in Lao PDR are highly critical of health system performance [2,21]. Third, nonadherence to the intervention due to unexpected events—such as technical issues with the website or not receiving the URL—may also bias study findings. To address this, we will assess participants’ frequency of website use in the endline survey and document any website-related downtime. Fourth, because all participants reside in the same 2 districts and the study uses an open-label design, cross-arm exposure and spillover effects may bias the results. To minimize these risks, we have separated the roles of staff: the team member responsible for communicating with the intervention group (sending biweekly reminders and links to the ratings website) is not involved in scheduling or conducting endline surveys or other administrative assessment activities. Additionally, to assess aggregate exposure, we will track daily traffic to the website. To measure exposure at the individual level, all participants will be asked at endline about their knowledge of the intervention, whether they received the link, how they received it, and how frequently they accessed the website. Finally, the researcher responsible for data analysis will also be involved in data collection; therefore, complete blinding of the analyst is not possible. In practice, however, the analyst will remain blinded during the endline assessments.

### Conclusions

In conclusion, if this demand-side intervention proves effective, it could offer a cost-effective approach for policy makers to ignite demand for high-quality care. To our knowledge, this is the first trial to examine how information about health care providers influences care-seeking decisions. Through this study, we aim to gain insights into patients’ decision-making regarding treatment locations and to explore the factors that shape these choices. We hope to extend our research findings to other populations, including adults, older adults, and individuals living with noncommunicable diseases. We recognize that care-seeking behaviors in Lao PDR differ from other settings due to variations in facility availability, cost, and quality of care. While our study is tailored to the health care landscape in Lao PDR, the findings may offer insights relevant to understanding the experiences of individuals seeking care in other contexts.

## Appendix

Multimedia Appendix 1 Baseline survey.

Multimedia Appendix 2 Endline survey.

Multimedia Appendix 3 CONSORT-eHEALTH checklist (V 1.6.1).

Multimedia Appendix 4 Informed consent form.

## Abbreviations

Lao PDR: Lao People’s Democratic Republic
NECHR: National Ethics Committee for Health Research
ODK: Open Data Kit
VITERBI: Vientiane Multigenerational Birth Cohort

## References

[1] Kruk ME, Gage AD, Arsenault C, Jordan K, Leslie HH, Roder-DeWan S, Adeyi O, Barker P, Daelmans B, Doubova SV, English M, García-Elorrio E, Guanais F, Gureje O, Hirschhorn LR, Jiang L, Kelley E, Lemango ET, Liljestrand J, Malata A, Marchant T, Matsoso MP, Meara JG, Mohanan M, Ndiaye Y, Norheim OF, Reddy KS, Rowe AK, Salomon JA, Thapa G, Twum-Danso NAY, Pate M (2018). High-quality health systems in the Sustainable Development Goals era: time for a revolution. The Lancet Global Health.

[2] Aryal A, Clarke-Deelder E, Phommalangsy S, Kounnavong S, Fink G (2024). Health system utilization and perceived quality among adults in Lao PDR: evidence from a nationally representative phone survey. BMC Public Health.

[3] (2018). Delivering quality health services: a global imperative for universal health coverage. World Bank.

[4] Croke K, Thapa Gk, Aryal A, Pokhrel S, Kruk Me (2023). The politics of health system quality: how to ignite demand. BMJ.

[5] Arrow K (2001). Uncertainty and the welfare economics of medical care. 1963. J Health Polit Policy Law.

[6] Dranove D, Kessler D, McClellan M, Satterthwaite M (2002). Is more information better? The effects of 'report cards' on health care providers (NBER working paper series no 8697). National Bureau of Economic Research.

[7] Jin G, Sorensen A (2006). Information and consumer choice: the value of publicized health plan ratings. J Health Econ Mar.

[8] Han X, Du Jt, Zhang T, Han W, Zhu Q (2021). How online ratings and trust influence health consumers’ physician selection intentions: an experimental study. Telematics and Informatics.

[9] Grabner-Kräuter S, Waiguny MKJ (2015). Insights into the impact of online physician reviews on patients' decision making: randomized experiment. J Med Internet Res.

[10] Lewis T, McConnell M, Aryal A, Irimu G, Mehata S, Mrisho M, Kruk Me (2023). Health service quality in 2929 facilities in six low-income and middle-income countries: a positive deviance analysis. The Lancet Global Health.

[11] Fink G, Kandpal E, Shapira G (2022). Inequality in the quality of health services: wealth, content of care, and the price of antenatal consultations in the Democratic Republic of Congo. Economic Development and Cultural Change.

[12] Wolf MS, Smith SG, Pandit AU, Condon DM, Curtis LM, Griffith J, O’Conor R, Rush S, Bailey SC, Kaplan G, Haufle V, Martin D (2018). Development and validation of the Consumer Health Activation Index. Med Decis Making.

[13] World Health Organization (WHO) (2005). Handbook: IMCI Integrated Management of Childhood Illness.

[14] Lewis TP, Kapoor NR, Aryal A, Bazua-Lobato R, Carai S, Clarke-Deelder E, Croke K, Dayalu R, Espinoza-Pajuelo L, Fink G, Garcia PJ, Garcia-Elorrio E, Getachew T, Jarhyan P, Kassa M, Kim SA, Mazzoni A, Medina-Ranilla J, Mohan S, Molla G, Moshabela M, Naidoo I, Nzinga J, Oh J, Okiro EA, Prabhakaran D, Roberti J, SteelFisher G, Taddele T, Tadele A, Wang X, Xu R, Leslie HH, Kruk ME (2023). Measuring people's views on health system performance: design and development of the People's Voice Survey. PLoS Med.

[15] Beatty PC, Willis GB (2007). Research synthesis: the practice of cognitive interviewing. Public Opinion Quarterly.

[16] People's Voice Survey Questionnaire. QuEST (Quality Evidence for Health System Transformation).

[17] WhatsApp encryption overview: technical white paper. WhatsApp.

[18] Andaleeb SS (2001). Service quality perceptions and patient satisfaction: a study of hospitals in a developing country. Soc Sci Med.

[19] Aryal A, Clarke-Deelder E, Afriyie D, Phommalangsy S, Fink G (2025). Factors influencing healthcare facility selection in an urban setting in Lao PDR: findings from a qualitative study. SSM - Health Systems.

[20] Croke K, Moshabela M, Kapoor NR, Doubova SV, Garcia-Elorrio E, HaileMariam D, Lewis TP, Mfeka-Nkabinde GN, Mohan S, Mugo P, Nzinga J, Prabhakaran D, Tadele A, Wright KD, Kruk ME (2024). Primary health care in practice: usual source of care and health system performance across 14 countries. The Lancet Global Health.

[21] Lewis TP, Kassa M, Kapoor NR, Arsenault C, Bazua-Lobato R, Dayalu R, Fink G, Getachew T, Jarhyan P, Lee H, Mazzoni A, Medina-Ranilla J, Naidoo I, Tadele A, Kruk ME (2024). User-reported quality of care: findings from the first round of the People's Voice Survey in 14 countries. The Lancet Global Health.

