# Supplementary material for: Informing Facility Selection Through a Web-Based User Ratings System: Protocol for a Randomized Controlled Trial Among Mothers in Urban Lao People’s Democratic Republic
Source: JMIR Res Protoc. 2025 Sep 4;14:e66085. doi: 10.2196/66085 (PMC12447015; doi:10.2196/66085)
Supplement: Multimedia Appendix 2 [file resprot_v14i1e66085_app2.pdf]

Endline survey (Conducted in-person)

Laos health facility user-rating study

Interviewer information

**Variable Name**	**Question Text**	**Response Options**	**Logic**
int	[Select your name from list]		This question is required.
id	[Select ID from the tracking sheet]		This question is required.

Introduction

Variable Name	Question Text	Response Options	Logic
	INTERVIEWER READ OUT:<br>SABAIDEE. My name is [YOUR NAME] and I work for Lao Public Health Institute which is part of Ministry of Health. Thank you for participating in this research. This will be my last visit for this study. May I continue in Lao?	1: Yes<br>2: No<br>99: Refused [DO NOT READ]	This question is required.
	INTERVIEWER READ OUT: Thank you for your time. If you want to reach our research team later on, you can call the Lao Tropical and Public Health Institute at 020 55 679 603		If NO or REFUSED for the above

Basic demographics

Variable Name	Question Text	Response Options	Logic
note_demographics	INTERVIEWER READ OUT:<br>I am going to start with some questions about you and your health.		
q1	1. Please tell me your age.		This question is required.
q2	INTERVIEWER READ OUT:<br>2. Could you please tell me if you are…?	1: <18<br>2: 18-29<br>3: 30-39<br>4: 40-49<br>5: 50-59<br>6: 60-69<br>7: 70-79<br>8: >80<br>99: Refused<br>[DO NOT READ]	Only if precise age is unknown in q1
	INTERVIEWER READ OUT: I am sorry we are only allowed to enrol participants 18 or older.		Read only if individual is less than 18
q3	3. What is your ethnicity?	1: Lao-Tai<br>2: Mon-Khmer<br>3: Hmong-Mien<br>4: Chinese-Tibetan<br>5: Other<br>6: Don't Know<br>99: Refused/Missing<br>[DO NOT READ]	This question is required.
q3.1	[Enter OTHER ethnicity]		If OTHER in q3

Variable Name	Question Text	Response Options	Logic
q5.1	5.1 What district do you live in?	Select from list	This question is required.
q5.11	[Enter OTHER district]		If OTHER in q5.1
q5.2	5.2 What village do you live in?	Select from list	This question is required.
q6	6. What is the highest level and grade or year of education that you have completed?	1.0: None; 2.0: Primary (primary 1-5 years); 3.0: Lower secondary (secondary 1-4 years); 4.0: Upper secondary (secondary 5-7 years); 5.0: Post-secondary and Non-tertiary (13-15 years); 6.0: Tertiary (Associates and higher); 99.0: Refused [DO NOT READ]	This question is required.
q7	7. How many children do you have?		This question is required.
q8	8. Is the child at or below 2.5 years old (30 months)?	1 Yes<br>2 No<br>99 Refused [DO NOT READ]	This question is required. Repeat for each child under 2 years old
q8.1	8.1 How many are below 2.5 years old (30 months)?		This question is required.
q9	9. How old is your child in months?		This question is required.
q10	10. What is the gender of your child?	1 Male<br>2 Female<br>3 Other<br>99 Refused [DO NOT READ]	This question is required.

Variable Name	Question Text	Response Options	Logic
q11	11. Does your child have health insurance? For example, you or your family has purchased or your employer has purchased for you?	1 Yes<br>2 No<br>99 Refused [DO NOT READ]	This question is required.
q12	12. What type of health insurance does your child have? Please tell us the primary type of health insurance you use if you have more than one.	1 National Health Insurance<br>2 Government - social security<br>3 Community-based insurance<br>4 Private insurance health insurance<br>5 No insurance<br>99 Refused [DO NOT READ]	This question is required.
q13	13. How would you describe the overall health status of your child?	1 Excellent<br>2 Very good<br>3 Good<br>4 Fair<br>5 Poor<br>99 Refused [DO NOT READ]	This question is required.
q14	14. Are you the person responsible for managing overall health of your child?	1 Yes<br>2 No<br>99 Refused [DO NOT READ]	This question is required.
q15	15 Please specify other person responsible for managing your child's health	TEXT	If NO or DO NOT KNOW in q14

Non-use of care

Variable Name	Question Text	Response Options	Logic
q16	16. In the past 3 months, was there a time when your child had a health problem and needed medical attention, but you did not get healthcare from a provider?	1 Yes<br>2 No<br>99 Refused [DO NOT READ]	This question is required.
q16_1	16.1 The last time this happened, what was the main reason?	1 High cost (e.g., high out of pocket payment, not covered by insurance)	If YES in q16

Variable Name	Question Text	Response Options	Logic
		2 Far distance (e.g., too far to walk or drive, transport not readily available)<br>3 Long waiting time (e.g., long line to access facility, long wait for the provider)<br>4 Poor healthcare provider skills (e.g., spent too little time with patient, did not conduct a thorough exam)<br>5 Staff don't show respect (e.g., staff is rude, impolite, dismissive)<br>6 Medicines and equipment are not available (e.g., medicines regularly out of stock, equipment like X-ray machines broken or unavailable)<br>7 Illness not serious enough<br>8 Went to the pharmacy<br>9 Do not know anyone to support me<br>10 Fear of infections<br>11 Other, specify<br>99 Refused [DO NOT READ]	
q16_2	[Enter reason for not using health care provider]	TEXT	If OTHER in q16_1

Parental health literacy and activation

Variable Name	Question Text	Response Options	Logic
	INTERVIEWER READ OUT:<br>For the next set of questions, please respond with Strongly Disagree, Disagree, Unsure, Agree, Strongly Agree. We will ask you questions about health care provider, we mean either doctor or a nurse.		

Variable Name	Question Text	Response Options	Logic
q17	17. I am comfortable discussing my children's health issues with health care providers	1: Strongly Disagree<br>2: Disagree<br>3: Unsure<br>4: Agree<br>5: Strongly Agree<br>99: Refused [DO NOT READ]	This question is required.
q18	18. I would talk to my provider if I feel that a wrong treatment is prescribed for my child	1: Strongly Disagree<br>2: Disagree<br>3: Unsure<br>4: Agree<br>5: Strongly Agree<br>99: Refused [DO NOT READ]	This question is required.
q19	19. I am comfortable expressing my concerns to my child's provider	1: Strongly Disagree<br>2: Disagree<br>3: Unsure<br>4: Agree<br>5: Strongly Agree<br>99: Refused [DO NOT READ]	This question is required.
q20	20. I always know how to help my child feel better when he or she is sick	1: Strongly Disagree<br>2: Disagree<br>3: Unsure<br>4: Agree<br>5: Strongly Agree<br>99: Refused [DO NOT READ]	This question is required.
q21	21. I always know when to take my child to a health care provider	1: Strongly Disagree<br>2: Disagree<br>3: Unsure<br>4: Agree<br>5: Strongly Agree<br>99: Refused [DO NOT READ]	This question is required.
q22	22. I always know what steps to take when my child has a health problem	1: Strongly Disagree<br>2: Disagree<br>3: Unsure	This question is required.

Variable Name	Question Text	Response Options	Logic
		4: Agree<br>5: Strongly Agree<br>99: Refused [DO NOT READ]	
q23	23. I always know where to look for information before making decisions about my child's health	1: Strongly Disagree<br>2: Disagree<br>3: Unsure<br>4: Agree<br>5: Strongly Agree<br>99: Refused [DO NOT READ]	This question is required.
q24	24. It is very easy for me to understand my child's health care provider's instructions	1: Strongly Disagree<br>2: Disagree<br>3: Unsure<br>4: Agree<br>5: Strongly Agree<br>99: Refused [DO NOT READ]	This question is required.
q25	25. It is very easy for me to ask my child's provider questions	1: Strongly Disagree<br>2: Disagree<br>3: Unsure<br>4: Agree<br>5: Strongly Agree<br>99: Refused [DO NOT READ]	This question is required.
q26	26. I can always take care of my child	1: Strongly Disagree<br>2: Disagree<br>3: Unsure<br>4: Agree<br>5: Strongly Agree<br>99: Refused [DO NOT READ]	This question is required.
q27	27. I am able to identify the best place to get care for my child when he/she is sick..	1: Strongly Disagree<br>2: Disagree<br>3: Unsure<br>4: Agree<br>5: Strongly Agree<br>99: Refused [DO NOT READ]	This question is required.

Variable Name	Question Text	Response Options	Logic
q28	28. My child will receive approximately the same care at any health facility we go to.	1: Strongly Disagree<br>2: Disagree<br>3: Unsure<br>4: Agree<br>5: Strongly Agree<br>99: Refused [DO NOT READ]	This question is required.
q29	29. My child's recovery time will be different depending on the health care provider I bring him/her to	1: Strongly Disagree<br>2: Disagree<br>3: Unsure<br>4: Agree<br>5: Strongly Agree<br>99: Refused [DO NOT READ]	This question is required.
q30	30. Our waiting time will be different depending on the health care provider I bring my child to.	1: Strongly Disagree<br>2: Disagree<br>3: Unsure<br>4: Agree<br>5: Strongly Agree<br>99: Refused [DO NOT READ]	This question is required.

Usual source of care

Variable Name	Question Text	Response Options	Logic
q31	31. If your child is sick tomorrow with mild fever mild fever of 38 and cough, which hospital, clinic or health center will you take your child to?	1: GOV_hospital;<br>2: GOV_health center;<br>3:PRIVATE_hospital;<br>4: PRIVATE_clinic<br>99: Refused<br>[DO NOT READ]	This question is required.
q32	32. [Select facility]	Select facility from list	This question is required.

Variable Name	Question Text	Response Options	Logic
q32.1	[Enter name of facility]	TEXT	If facility is OTHER in q32
q34	34. What district is this FACILITY in?	1. Chanthabuly<br>2. Sikhottabong<br>3. Xaysetha<br>4. Sisattanak<br>5. Naxaithong<br>6. Xaythany<br>7. Hadxaifong<br>8. Sangthong<br>9. Mayparkngum<br>99. Refused [DO NOT READ]	This question is required.
q35	35. What village is this FACILITY in?	SELECT FROM LIST	This question is required.
q35.1	35.1 [Enter Other village]		If village is OTHER in q35
q36	36. Why did you choose this FACILITY? Please tell us the main reason.	1 Low cost<br>2 Short distance<br>3 Short waiting time<br>4 Good healthcare provider skills<br>5 Staff shows respect<br>6 Medicines and equipment are available<br>7 Only facility available<br>8 Covered by insurance<br>9 Know someone at the facility<br>10 Other<br>99 Refused [DO NOT READ]	This question is required.
q36.2	[Enter OTHER reason]	TEXT	If OTHER in q36

Variable Name	Question Text	Response Options	Logic
q36.3	36.3 Was this choice informed by online or digital information services?	1 Yes<br>2 No<br>99 Refused [DO NOT READ]	This question is required.
q37	37. If your child is sick tomorrow with a very high fever of 39-40 and a red rash covering their body, which hospital, clinic or health center will you take your child to?	1: GOV_hospital<br>2: GOV_health center<br>3: PRIVATE_hospital<br>4: PRIVATE_clinic	This question is required.
q38	38. [Select facility]	Select from LIST	This question is required.
q38.1	[Enter name of facility]	TEXT	If OTHER in q38
q41	41. What district is this FACILITY in?	Select form list of districts in Vientiane capital	This question is required.
q42	42. What village is this FACILITY in?	Select form respective district	This question is required.
q42.1	42.1 [Enter Other village]	TEXT	If OTHER in q42
q43	43. Why did you choose this FACILITY? Please tell us the main reason.	1 Low cost<br>2 Short distance<br>3 Short waiting time<br>4 Good healthcare provider skills<br>5 Staff shows respect<br>6 Medicines and equipment are available<br>7 Only facility available<br>8 Covered by insurance<br>9 Know someone at the facility<br>10 Other, specify<br>99 Refused [DO NOT READ]	This question is required.
q43.1	[Enter OTHER reason]	TEXT	If OTHER in q43

Most recent visit for a sick child

Variable Name	Question Text	Response Options	Logic
	INTERVIEWER READ OUT:<br>We would like to know about your use of health services of your children less than 2 years old. Please answer only about your own experience.		
q44	44. When is the last time you visited a hospital, clinic or health center for your child?	1. <1 month<br>2. 2-3 months<br>3. 4-6 months<br>4. I did not take my child for care in the past 6 months<br>99. Refused [DO NOT READ]	This question is required.
q45.1	45.1 Did your child have cough?	1 Yes<br>2 No<br>99 Refused [DO NOT READ]	If sick in the last 3 months
q45.2	45.2 Did your child have diarrhea?	1 Yes<br>2 No<br>99 Refused [DO NOT READ]	If sick in the last 3 months
q45.3	45.3 Did your child have fever?	1 Yes<br>2 No<br>99 Refused [DO NOT READ]	If sick in the last 3 months
q45.4	45.4 Did your child have ear problems?	1 Yes<br>2 No<br>99 Refused [DO NOT READ]	If sick in the last 3 months
q45.5	45.5 Did your child have vomitting?	1 Yes<br>2 No<br>99 Refused [DO NOT READ]	If sick in the last 3 months
q45.6	45.6 Did your child have injuries?	1 Yes<br>2 No<br>99 Refused [DO NOT READ]	If sick in the last 3 months
q45.7	45.7 Did your child have skin rash?	1 Yes<br>2 No<br>99 Refused [DO NOT READ]	If sick in the last 3 months

Variable Name	Question Text	Response Options	Logic
q46.1	q46.1 Was your child unable to drink or breastfeed?	1 Yes<br>2 No<br>99 Refused [DO NOT READ]	If sick in the last 3 months
q46.2	q46.2 Did your child vomit everything?	1 Yes<br>2 No<br>99 Refused [DO NOT READ]	If sick in the last 3 months
q46.3	q46.3 Did your child have convulsions?	1 Yes<br>2 No<br>99 Refused [DO NOT READ]	If sick in the last 3 months
q46.4	q46.4 Was your child lethargic or unconscious	1 Yes<br>2 No<br>99 Refused [DO NOT READ]	If sick in the last 3 months
q47	47. What is the name of the hospital, clinic or health center you visited?	1.0: GOV_hospital<br>2.0: GOV_health center<br>3.0: PRIVATE_hospital<br>4.0: PRIVATE_clinic	Show this question only if: ${q44}<4; This question is required.
q48	48. [Select facility]	SELECT from LIST	Show this question only if: ${q44}<4; This question is required.
q48.1	48.1 [Enter name of facility]	TEXT	Show this question only if: ${q48}=80 or ${q48}=81 or ${q48}=82 or ${q48}=83 ; This question is required.
q50	50. What district is this FACILITY in?	Select form list of districts in Vientiane capital	This question is required.

Variable Name	Question Text	Response Options	Logic
q51	51. What village is this FACILITY in?	Select form respective district	This question is required.
q51.1	51.1 [Enter Other village]	TEXT	If OTHER in q51
q52	52. Why did you choose this FACILITY? Please tell us the main reason.	1. Low cost<br>2. Short distance<br>3. Short waiting time<br>4. Good healthcare provider skills<br>5. Staff shows respect<br>6. Medicines and equipment are available<br>7. Only facility available<br>8. Covered by insurance<br>9. Know someone at the facility<br>10. Other, specify<br>99. Refused [DO NOT READ]	
q52.1	[Enter OTHER reason]		If OTHER in q52

Care experience

Variable Name	Question Text	Response Options	Logic
q53	53. Once you arrived at the facility, approximately how long did you and your child wait before seeing the provider?<br><br>Please do not include wait time for other parts of the visit such as lab tests, X-rays, or trips to the pharmacy.	1. < half hour<br>2. 30 - 60 minutes (0.5 - 1 hour)<br>3. 61 - 120 minutes (1.01 - 2 hours)<br>4. 121 - 180 minutes (2.01 - 3 hours)<br>5. 181 - 240 minutes (3.01 - 4 hours)<br>6. 241 - 300 minutes (4.01 - 5 hours)<br>7. 301 - 360 minutes (5.01 - 6 hours)<br>99. Refused [DO NOT READ]	This question is required.

Variable Name	Question Text	Response Options	Logic
q54	54. Once you saw the healthcare provider, approximately how long did he or she spend with you and your child?	1. < 10 minutes<br>2. 11 - 20 minutes<br>3. 21 - 30 minutes<br>4. 31 - 40 minutes<br>5. 41 - 50 minutes<br>6. 51 - 60 minutes<br>99. Refused [DO NOT READ]	This question is required.

Perceived quality of the most recent visit

Variable Name	Question Text	Response Options	Logic
	[INTERVIEWER READ OUT]: Thinking about the quality of care for your child received at THE FACILITY (Q48), how would you rate the following…?		
q55	55. How would you rate the overall quality of care you received?	1. Excellent<br>2. Very good<br>3. Good<br>4. Fair<br>5. Poor<br>99. Refused [DO NOT READ]	This question is required.
q56	56. How would you rate the knowledge and skills of your provider?	1. Excellent<br>2. Very good<br>3. Good<br>4. Fair<br>5. Poor<br>99. Refused [DO NOT READ]	This question is required.
q57	57.How would you rate the equipment and supplies that the provider had available, such as medical equipment or access to lab tests?	1. Excellent<br>2. Very good<br>3. Good<br>4. Fair<br>5. Poor	This question is required.

Variable Name	Question Text	Response Options	Logic
		99. Refused [DO NOT READ]	
q58	58. How would you rate the level of respect your provider showed you?	1. Excellent<br>2. Very good<br>3. Good<br>4. Fair<br>5. Poor<br>99. Refused [DO NOT READ]	This question is required.
q59	59. How would you rate whether your provider knew about your prior visits and test results?	1. Excellent<br>2. Very good<br>3. Good<br>4. Fair<br>5. Poor<br>99. Refused [DO NOT READ]	This question is required.
q60	60. How would you rate whether your provider explained things in a way you could understand?	1. Excellent<br>2. Very good<br>3. Good<br>4. Fair<br>5. Poor<br>99. Refused [DO NOT READ]	This question is required.
q61	61. How would you rate whether your provider involved you as much as you wanted to be in decisions about your care?	1. Excellent<br>2. Very good<br>3. Good<br>4. Fair<br>5. Poor<br>99. Refused [DO NOT READ]	This question is required.
q62	62. How would you rate the amount of time your provider spent with you?	1. Excellent<br>2. Very good<br>3. Good<br>4. Fair<br>5. Poor<br>99. Refused [DO NOT READ]	This question is required.

Variable Name	Question Text	Response Options	Logic
q63	63. How would you rate the amount of time you waited before being seen?	1. Excellent<br>2. Very good<br>3. Good<br>4. Fair<br>5. Poor<br>99. Refused [DO NOT READ]	This question is required.
q64	64. How would you rate the courtesy and helpfulness of the healthcare facility staff, other than your provider?	1. Excellent<br>2. Very good<br>3. Good<br>4. Fair<br>5. Poor<br>99. Refused [DO NOT READ]	This question is required.
q64.1	64.1 How would you rate the cost of health care services?	1. Excellent<br>2. Very good<br>3. Good<br>4. Fair<br>5. Poor<br>99. Refused [DO NOT READ]	This question is required.
q64.2	64.2 How would you rate the cleanliness?	1. Excellent<br>2. Very good<br>3. Good<br>4. Fair<br>5. Poor<br>99. Refused [DO NOT READ]	This question is required.

Endorsement of clinic

Variable Name	Question Text	Response Options	Logic
q65	Using a scale from 1 to 10, where 1 means you definitely would not recommend and 10 means you definitely would recommend, how likely is it	1. 1<br>2. 2<br>3. 3	This question is required.

Variable Name	Question Text	Response Options	Logic
	that you would recommend this healthcare provider or facility to a friend or family member?	4. 4<br>5. 5<br>6. 6<br>7. 7<br>8. 8<br>9. 9<br>10. 10	

Expectation of quality

Variable Name	Question Text	Response Options	Logic
	INTERVIEWER READ OUT:<br>Now I would like to read you a story to get an understanding of how you rate the quality of care Ms Noy or Mr Noy has been feeling increasing stomach pain for the past 3 days and decides to go to clinic.		
q66	66. At the health facility, the doctor does not ask about his symptoms or examine his body; the doctor gives him (her) pain medication and does not give him (her) the diagnosis. How would you rate the quality of care provided?<br><br>Please take everything into account and give us your honest assessment.	1. Excellent<br>2. Very good<br>3. Good<br>4. Fair<br>5. Poor<br>99. Refused [DO NOT READ]	This question is required.
q67	67. Now Ms Noy or Mr Noy goes to another clinic. There the doctor examines him (her) and orders a blood test. He (She) tells him it is not serious, advises a light diet, and asks him (her) to come back if it gets worse. How would you rate the quality of care provided?	1. Excellent<br>2. Very good<br>3. Good<br>4. Fair<br>5. Poor<br>99. Refused [DO NOT READ]	This question is required.

Closing Section

Variable Name	Question Text	Response Options	Logic
	INTERVIEWER READ OUT: Thank you for taking part in this study. I have only a few remaining questions. The answers given by you are confidential and will not be linked back to you. We appreciate your time.		
q85	85. Are you aware of a webpage that provides information on hospitals, clinics and health centers and scores them?	1. Yes<br>2. No<br>99. Refused [DO NOT READ]	This question is required.
q86	86. Did you receive a link to the webpage that provides information on hospitals, clinics and health centers and scores them?	1. Yes<br>2. No<br>99. Refused [DO NOT READ]	If aware of the webpage in q85
q87	87. How often did you access the webpage that provides information on hospitals, clinics and health centers and scores them?	1. At least once per day<br>2. At least once per week<br>3. At least once per month<br>4. Once or twice in the previous 3 months<br>5. I did not access the webpage<br>99. Refused [DO NOT READ]	If aware of the webpage in q85
q88	88. Did you think the webpage consisting of ratings of hospitals, clinics and health centers was Excellent, Very good, Good, Fair or Poor?	1. Excellent<br>2. Very good<br>3. Good<br>4. Fair<br>5. Poor<br>99. Refused [DO NOT READ]<br>1. At least once per day<br>2. At least once per week<br>3. At least once per month<br>4. Once or twice in the previous 3 months<br>5. I did not access the webpage<br>99. Refused [DO NOT READ]	If aware of the webpage in q85

Variable Name	Question Text	Response Options	Logic
q89	89. What did you like about the webpage consisting of ratings of hospitals, clinics and health center?		If aware of the webpage in q85
q90	90. What did you dislike about the webpage consisting of ratings of hospitals, clinics and health centers?		If aware of the webpage in q85
q91	91. During the last interview, you told us that you have access to a smartphone. Do you share that smartphone with your partners or other relatives?	1. Yes<br>2. No<br>99. Refused [DO NOT READ]	If aware of the webpage in q85
	INTERVIEWER READ OUT:<br><br>Thank you for taking part in this study. We appreciate your time.		
