# Supplementary material for: Informing Facility Selection Through a Web-Based User Ratings System: Protocol for a Randomized Controlled Trial Among Mothers in Urban Lao People’s Democratic Republic
Source: JMIR Res Protoc. 2025 Sep 4;14:e66085. doi: 10.2196/66085 (PMC12447015; doi:10.2196/66085)
Supplement: Multimedia Appendix 4 [file resprot_v14i1e66085_app4.pdf]

Swiss TPH
Swiss Tropical and Public Health Institute

Informed Consent Form

Written Informed Consent Form for the participation in a study
Please read this form carefully. Please ask if you do not understand or want to know something. Your written consent is required for study participation.

**Study title (scientific and colloquial)**:	Mobile phone intervention to improve satisfaction with health care (Laos Mobile Ratings, Version 1.0, 17.08.2023)
**Responsible institution (sponsor with address)**:	Lao Tropical and Public Health Insitute (Lao TPHI)<br>Vientaine, Lao People's Democratic Republic<br><br>Swiss Tropical and Public Health Institute (Swiss TPH)<br>Kreuzstrasse 2, 4123 Allschwil, Switzerland
**Site of study conduct**:	Vientiane, Lao People's Democratic Republic
Study investigator**:**<br>Name and first name in capital letters:<br>Role in the study:	**Mr. AMIT ARYAL**<br>Principal Investigator<br>amit.aryal@swisstph.ch<br><br>**Dr. SENGCHANH KOUNNAVONG**<br>Local Project Partner<br>sengchanhkounnavong@hotmail.com
**Participant:**<br>Name and first name in capital letters:	
**Date of birth (DD/MM/YY)**	
**Sex**	☐ female ☐ male
**Can the participant read?**	☐ yes ☐ no
**Does the participant have exclusive access to a mobile phone?**	☐ yes ☐ no

Introduction: Despite increasing options for public and private health care providers in Laos, choosing a high-quality health provider or a facility is difficult because timely and reliable information about providers is not readily available. People rely on social networks or previous experiences to select providers. However, in Laos, only 28% describe their recent visit to a health care provider as high-quality suggesting that while there are increasing options for care, people may need support to find providers that meet their quality needs.

Rapid adoption of mobile phones in Laos, particularly in urban areas, offer opportunities to enhance people's access to timely quality information about health care providers. We will use mobile phones to collect and disseminate quality information about providers – known to be valued by Laotians – to improve their access to quality care as well as their overall satisfaction with care.

Purpose of the study: The purpose of this study is to assess whether routinely collected information on quality of care received can improve access to quality care and patient satisfaction with care.

Personal participation: You are free to decide whether you will be involved in the study. You are only eligible for this study if you are a woman, live in Chantabouly or Sikhottabongm, you are at least 18 years old with at least one child less than 2 years old, have access to a smart phone, use WhatsAPP and can read and write.

Study procedure: If you agree to participate in this study, we will ask you to spend about 60 minutes with me today. I will ask you about health care providers for your child. I will ask you about health care providers you visited most recently, when and what you think about high quality health care. I will then show you how the types of mobile messages you will receive from us and how to respond to them. You are expected to respond mobile messages every 2 weeks for a total of 6 times during the study period. The study period will last for 3 months from today. We will visit you again at the end of the study period – 3 months from today – and ask you similar questions about health care for your child.

Risks / Benefits: While there is a potential risk of breach of privacy, this risk is minimal because the questions asked in the study are not highly sensitive and we will treat your personal information confidentially. When answering questions about your experiences with the health care, there is a risk you may experience emotional distress. However, we anticipate this distress would be mild, and short-lived. The benefits of this study are that it provides an opportunity to study how information about quality of providers can influence patient satisfaction with care.

Confidentiality: Your personal information will be treated confidentially and will be accessible to only the researchers directly involved with this study. The list of study participants will be stored separately at a secure place, which will be protected from access of unauthorized staff.

Participation / withdrawal: Your participation in this study is entirely voluntary. You are free to refuse or withdraw your consent at any time without any further obligation. If you decide to withdraw from the study, we may use the information you have provided us up to that point.

Costs / compensation: You will not receive any compensation for participating in this study.Your involvement to this study will not result in any additional costs. The study is funded by the Swiss Tropical and Public Health Institute (Swiss TPH).

Future use of data: After publication and dissemination of the results, all personal information will be removed from the data; fully de-identified data will be saved on Swiss TPH servers for at least 15 years and be made available after study completion.

Contact person for questions: If you have questions, you can ask the study fieldworkers who will attempt to answer or transfer them to one of the main researchers. You can also contact the head of the research team in Laos, Dr Somphou, via email at sengchanhkounnavong@hotmail.com. You can also contact the Swiss research team via email at amit.aryal@swisstph.ch

The participant will receive a copy of the Informed consent

- I was informed verbally and in writing by the undersigned study staff member about the purpose, the course of the study about possible advantages and disadvantages as well as about possible risks.
- I voluntarily participate in this study and accept the content of the written information provided. I had enough time to make my decision.
- My questions regarding participation in this study have been answered. I retain the written information and receive a copy of my signed informed consent.
- I agree that the responsible experts of the sponsor, the responsible ethics committee and the Swiss TPH and the Lao Tropical and Public Health Institute may inspect my unencrypted data for review and control purposes, but with strict maintenance of confidentiality.

- I can withdraw from the study at any time and without giving reasons. The data and samples collected up to the time of withdrawal will be used for the evaluation of the study.
- I am aware that the obligations stated in the participant information must be complied with. In the interest of my health, the project leader may exclude me from the study at any time.

Written Informed Consent Form for the participation in a study

Please read this form carefully. Please ask if you do not understand or want to know something.

Your written consent is required for study participation.

Name, age :<br><br>Location, date:	Participant’s signature or thumbprint:
Location, date:<br><br>Name of the witness:	Witness’s signature:

Declaration/statement of project employee: “I (project staff) state that I have explained in detail the project objectives, duration, activities, and benefits and risk for the participants. I have also explained the privacy of the information and all the other aspects described in this informed consent form. I have taken part of the entire consent interview and signature process.”

Location, date	Signature of the project staff member**:**<br><br>Name and first name in capital letters:<br><br>Role in the study: in capital letters
